# Connecticut Valley Dental Society

**Published:** 1887-10

**Authors:** Geo. A. Maxfield

**Affiliations:** Secretary


(TO BE CONTINUED.)

### CONNECTICUT VALLEY DENTAL SOCIETY.

SEMI-ANNUAL MEETING, HELD AT MONTREAL, P. Q., JULY 19 TO 21, INCLUSIVE.

REPORTED BY GEO. A. MAXFIELD, D. D. S., SECRETARY.

CONTINUED FROM PAGE 495.

### TUESDAY EVENING SESSION.

Dr. W. Geo. Beers, of Montreal, read a paper on "Alveolar Abscess." The essayist reviewed the literature of the subject, and detailed some of the views of early writers. He said that whatever the primeval cause of alveolar abscess, it always originates in the apical space, as the result of apical pericementitis. An old Montreal practitioner was the first to introduce arsenic to the profession for the devitalization of tooth-pulps, but Spooner should not be held responsible for its abuse. Many cases of abscess are due to the injudicious use of this drug. The old theory that it was in the course of time absorbed, even at the expense of the translucency of the enamel, satisfied the practitioners of the early school, but dead pulps, unlike dead men, do tell tales, and the effects of the use of arsenic have been too plainly revealed to us to doubt.

He said that in some cases constitutional treatment is indicated. In opening canals it is not safe to use drills where broaches will not penetrate. A root may be curved at the apex, and this may be discerned if a broach can be inserted, while a drill might do possible mischief. In the early stage of hyperæmia he recommended a kind of massage with the finger.

DISCUSSION.

*Dr. Atkinson*—When a case first presents I do not hesitate, after removing the pulp, to pass a drill through the root so as to relieve the congestion. If the tooth is painful to bite upon, with the engine cut down the occlusion. Many are not sharp enough to see that the occlusion is a cause of irritation. Often this is all that is necessary to be done, and after doing this you can send the patient home.

*Dr. Shepard*—What if the patient comes back with a worse pain ?

*Dr. Atkinson*—Tie a string tightly about the tooth and have the patient hold it; then with a scalpel cut through the gum over the root, right down to the tooth, and let it bleed freely; this relieves the patient instantly. Cut in straight lines down to the lower margin of the alveolar plate; do not cut below the margin; the object is depletion.

*Dr. Shepard*—Have you not then lanced the abscess ?

*Dr. Atkinson*—You are impressing what I do not say. The pericementum is connective tissue, and it is in this tissue at the apex of the root that the abscess forms. This connective tissue is a band about the root of the tooth, and we simply cut this band.

*Dr. Shepard*—You are not clear. Do you not give vent by making the opening ?

*Dr. Atkinson*—No ! Cutting the band relieves the tension. I do not care if I cut through to the dentine; it will heal by first intention. This band of connective tissue fiber, when inflamed, hugs tightly around the root. Often I have had patients exclaim, "that feels good !" when I have cut it in this way. It is tension about the root by the pericementum that causes the pain.

*Dr. Shepard*—But patients come back next day in worse condition.

*Dr. Atkinson*—They do not. I never have known a case that was not cured if properly treated. There is no such thing as not curing an abscess. A short time ago a dentist brought to me a case that had not yielded to his treatment, and it was only because he had not been sharp enough to diagnose it as an abscess in the antrum.

*Dr. Shepard*—Can one diagnose accurately enough to treat all cases successfully?

*Dr. Atkinson*—Those who diagnose cases clear through, every time, are not the ones to follow as your teachers. Know what step to take first, then watch developments.

*Dr. Shepard*—What is the next step to take?

*Dr. Atkinson*—Do not make a stroke in the dark.

*Dr. Shepard*—I want to know something more.

*Dr. Atkinson*—There is much more to be said on this subject. These abscesses are caused by dead pulps; after removing them, go through the gum from the outside, and with the engine bur slash around the end of the root; if necessary, cut off the end of the root.

*Dr. Bazin*—Will it make any difference if the patient is a hard drinker?

*Dr. Atkinson*—Not any difference; of course you must consider the habits of your patients. It will be necessary for you to have entire control. Abscesses can be entirely prevented, and the dentist is to blame who allows one to recur.

*Dr. Stowell*—This slashing around is quite painful to the patient. Is there any objection to injecting cocaine? I have, by its use, been quite successful many times in making this part of the operation a painless one.

*Dr. Atkinson*—We are not well enough acquainted with that drug. It may not be safe to use it in such close proximity to the brain. Do not notify your patients of what you are going to do.

*Dr. Maxfield*—Wouldn't you recommend painting the gum with carbolic acid before lancing?

*Dr. Atkinson*—Yes, and it then might be done almost painlessly.

Subject passed.

WEDNESDAY EVENING SESSION.

The meeting was called to order at 8 o'clock.

Dr. R. R. Andrews, of Cambridge, read a paper upon "Development of the Teeth," illustrated with the Stereopticon. He gave a detailed account of his manner of preparing the slides for the study. The specimens used were all prepared from embryo pigs, calves and cats. Placing these under the microscope, the camera is placed in position and a negative is taken; from the negative thus obtained a positive is taken, and this last is used as the slide in the lantern. Over fifty different slides were projected on the screen, showing the tooth in the different stages of its development, making one of the finest exhibitions ever given before a scientific body. Owing to insufficient light he was unable to bring out clearly a number of his finer specimens.

THURSDAY MORNING SESSION.

Called to order at 9 o'clock.

The discussion of Dr. Andrews' paper was declared in order.

*Dr. Shepard*—I regret very much that the light last night was not sufficiently strong to bring out all the fine details of the microphotographs. With a more powerful light all these would have been brought out very clearly.

*Dr. Barrett*—I want to say how much I appreciated Dr. Andrews' effort last evening. It was the finest display of its kind that I have ever seen. To be sure, some of the finer views were not as well brought out as we would like to have had them, yet enough was shown to make it a rich treat for every one of us. I feel greatly indebted to Dr. Andrews.

*Prof. Mills*—I am very much indebted for the demonstration and teaching of last night. I felt a good deal of sympathy for our friend, who has had to fight against established opinions for fifteen years. I think that if this work could be attacked from the light of the human embryo, it would be well. Those could be obtained if medical men were more interested. A rapid change of human teeth is going on. In the course of the evolution of man the jaws have changed, and there is less room for the teeth. Teeth are not infrequently absent, or appear late. This change in the development of the teeth has been related to nutritional changes, and that has not kept up with development of the brain, especially the cerebrum. A short time ago I wrote an article, which was published in the *Canadian Record of Science,* in which I tried to draw attention to the loss of the hair from a physiological standpoint. The hair and the teeth are dermal structures, which receive their blood supplies from small branches of arteries that anastomose at the carotids. It is possible that the non-appearance of the teeth at the proper time, and their early decay, have the same common origin with that of loss of the hair. I think that on this subject dentists might throw some light. The hair of women, however, has a persistent vitality not met with in men, so that in them the same relation to the teeth does not probably hold good.

*Dr. Black*—I acknowledge my obligations to Dr. Andrews for last night's work. The method of photo-micrography is a plan of exhibiting, through which many may be taught what cannot be taught by any of the ordinary plans of microscopic study. These pictures are not liable to tell lies, and more likely to tell the truth than those drawn with pencil. I agree with Prof. Mills in regard to what he says about the human embryo. Perhaps Dr. Andrews could also help us in another work. With his high powers and trained observation he may be able to project on the screen the different forms of micro-organisms, and then, perhaps, we may understand the difference between them, and divide them into species as easily as animals.

*Prof. Mayr*—I am sorry to see in so excellent a book as that of Dr. Heitzmann a passage which speaks slightingly of micro-photography. Drawings are, in some respects, untrue, and the finer and sharper they are drawn, the farther they are from the truth. This point appears most strikingly when we compare the beautiful (and as nearly truthful as possible) drawings in Dr. Heitzmann's work with the blurred outlines of excellent photographs. In the microscope we do not see sharp lines; we see blurred lines, and the photograph correctly copies this condition of affairs.

*Dr. Atkinson*—In regard to drawings as compared with photographs, I have to say that the best drawings of that master of graphic representations, Heitzmann, are in a sense lies; that is, the unimportant parts are held in abeyance, to bring out into high relief the parts that are desired to be brought out, and in that sense drawings are ahead of photographs. I protest against this manner of naming things that Dr. Black used in his paper. This employing Greek words that no one understands. Away with such things; let us use plain English that every one understands.

*Dr. Black*—I feel inclined to say a word in regard to the matter of talking Greek. If I should stand up before you and translate these Greek names, you would not know what I meant without an explanation. As the books employ the terms that I used, I thought it better to take them as set down rather than to attempt the invention of new ones, and inevitably to complicate our nomenclature yet more and more.

Subject passed.

